# Dietary Intake and Genetic Background Influence Vitamin Needs during Pregnancy

**DOI:** 10.3390/healthcare10050768

**Published:** 2022-04-21

**Authors:** Maria Paola Bertuccio, Monica Currò, Daniela Caccamo, Riccardo Ientile

**Affiliations:** Department of Biomedical Sciences, Dental Sciences and Morpho-Functional Imaging, University Hospital Polyclinic, 98125 Messina, Italy; mbertuccio@unime.it (M.P.B.); moncurro@unime.it (M.C.); dcaccamo@unime.it (D.C.)

**Keywords:** nutrient requirement, polymorphisms, pregnancy, toxicity, vitamin metabolism, vitamin supplementation

## Abstract

Numerous approaches demonstrate how nutritional intake can be sufficient to ensure the necessary supply of vitamins. However, it is evident that not all vitamins are contained in all foods, so it is necessary either to combine different food groups or to use a vitamin supplement to be well-fed. During pregnancy, deficiencies are often exacerbated due to increased energy and nutritional demands, causing adverse outcomes in mother and child. Micronutrient supplementation could lead to optimal pregnancy outcomes being essential for proper metabolic activities that are involved in tissue growth and functioning in the developing fetus. In order to establish adequate vitamin supplementation, various conditions should be considered, such as metabolism, nutrition and genetic elements. This review accurately evaluated vitamin requirements and possible toxic effects during pregnancy. Much attention was given to investigate the mechanisms of cell response and risk assessment of practical applications to improve quality of life. Importantly, genetic studies suggest that common allelic variants and polymorphisms may play an important role in vitamin metabolism during pregnancy. Changes in gene expression of different proteins involved in micronutrients’ metabolism may influence the physiological needs of the pregnant woman.

## 1. Introduction

In the field of functional homeostasis, vitamins play a relevant role in mineral metabolism and regulation of enzymatic activities, and are useful to prevent many pathological conditions. Although many observations lead to the belief that a balanced diet is the best practice to take the adequate dose of vitamins, numerous studies suggested that, in case of deficiency, the specific supplementation of a single vitamin can lead to a successful resolution of some pathophysiological conditions [1,2,3,4].

The biological function of vitamins is associated with different roles and the demand, to ensure a good state of health, shows a great inter-individual variability.

Consistent with availability of vitamins, different effects leading to a healthy status may be associated with various physiological conditions. For example, vitamin D, other than regulating bone metabolism, also plays extra-skeletal functions, including regulation of the immune system response [5], as well as antioxidant properties of different molecules, such as vitamin E, vitamin C, and beta-carotene, are able to protect biological systems from various stressors. Furthermore, vitamins B9 and B12 are involved in homocysteine metabolism and also play a pivotal role in the process of erythropoiesis [6], and vitamin C, other than being a powerful antioxidant, is essential for iron absorption [7].

On the basis of these multiple effects, it may be difficult to establish a range of vitamin plasma concentrations under physiological conditions. Moreover, vitamin requirement changes in different physiological conditions, such as pregnancy and the nutritional status of newborns. In addition, nutrient levels, which are considered adequate for growth, feed efficiency, gestation and breast feeding, may result in being insufficient to maintain a robust immune response and improve disease resistance [8,9]. Furthermore, there are physiological processes occurring in response to fetus development, such as changes in the endocrine, reproductive, cardiovascular, respiratory, and gastrointestinal systems.

The measurement of vitamin serum levels during pregnancy can be useful to establish if a supplementation is necessary. In fact, supplementation with multi-vitamin complexes is a common practice during pregnancy. Some results demonstrated that the intake of multi-vitamin complexes before pregnancy or during early pregnancy does not counteract the possibility of having a miscarriage, even if multi-vitamin complexes plus iron and folic acid reduced the risk of neonatal mortality. Further studies are needed to evaluate the effect of different combination of vitamins on miscarriage and abortion-related outcomes [10,11].

However, it must be taken into account that excessive vitamin nutrient supplementation can cause an intake beyond the tolerable upper intake level [12], increasing the risk of adverse events. Thus, pregnant women, and also the general population, should be advised not to exceed the intake of multivitamin preparations in the absence of a previous check-up of vitamin status. The reason for this caution is mostly related to the hormetic behaviour of lipophilic vitamins, which are effective at very small concentrations, like all vitamins, but can produce unwanted detrimental effects in case of elevated levels, as observed in hypervitaminosis resulting from excess supplementation [13].

Many studies on vitamin supplementation have also been conducted on animal and cellular models, leaving its relevance for possible effects in humans uncertain. Hence, this narrative review was aimed to emphasize studies on pregnant women by performing literature searches from 2000 to 2021 on PubMed, Google Scholar, and the Cochrane Library, choosing as key words vitamin, pregnancy and genetic polymorphism. Predominantly, the article shows the results of literature reviews, guidelines or research articles that explicitly indicate the strategy to ensure an adequate intake of some vitamins during pregnancy, also according to different genetic features.

**Figure 1:**
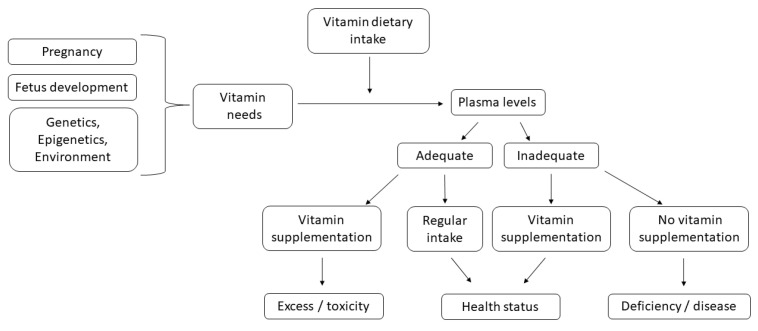
Flow chart to explore how individual susceptibility affects pregnancy health and outcomes. Vitamins must be ingested through diet or supplements and much attention should be paid to their adequate intake, which should be established as function of real needs.

In particular, the review focused on the consequences of inappropriate consumption or supplementation of some vitamins during pregnancy, particularly in the case of fat-soluble vitamins that tend to accumulate in body tissues leading to toxic effects [14,15,16]. We also examined the composite effects of genetic polymorphisms and dietary vitamin intake to assess whether genetic background influences nutrient vitamin requirement in pregnant women (Figure 1).

## 2. Relevant Findings on Fat-Soluble Vitamins

Available literature data on critical issues related to the supplementation of fat-soluble vitamins A, E, D, and K, in pregnant women are discussed in the following paragraphs.

Normal reference levels and recommended intake, when available, of each vitamin in different physiological conditions, have been derived from literature data, guidelines of the European Endocrinological Society, Dietary Reference Intakes (DRI) reports, and the Institutes of Medicine National Academy (see www.nap.edu accessed on 3 March 2022), and are shown in Table 1.

### 2.1. Vitamin A

Vitamin A is involved in several functions, such as normal immune and visual system function, epithelial integrity and red blood cell production [17,23,24], especially during pregnancy. Vitamin A affects the antibody response to T-cell-dependent antigens, which is also directly modulated by retinoic acid [25]. In addition, retinoid compounds, including all-trans-retinol, produce antioxidant effects, alone or in association with vitamin E and tocopherol derivatives [26].

The recommended dietary allowance (RDA) of vitamin A, that is, the average level of recommended daily dietary intake judged adequate by Food and Nutrition Board to meet the known nutrient needs of healthy individuals, can be met by the consumption of leafy green vegetables, orange and yellow vegetables and fruits (carrots, sweet potatoes, pumpkin, mango, cantaloupe), fish oil, eggs and beef liver, among other things (see www.nap.edu accessed on 3 March 2022).

Changes in the requirement of vitamin A are observed during pregnancy, with an increase of 10% in early pregnancy, and about 200% in late pregnancy, compared with normal requirement (Table 1). Vitamin A deficiency easily develops in early rather than in late pregnancy. Chen and colleagues observed that serum vitamin A levels were deficient in 24.98% of pregnant women examined [26].

Vitamin A deficiency increases the risk of miscarriage, night blindness, and pregnancy complication. A severe deficiency can cause fetal malformations affecting the embryonic development as described in a recent narrative review [18]. On the other hand, another review reported that high levels of vitamin A also increase birth defect risk [27]. Similarly, in a comparative study it has been shown that retinoids may be mainly responsible for toxic events [28]. In middle Europe, consuming a large amount of fish liver could be associated with toxic effects of retinoids assumption. The risk of excessive fish liver consumption during pregnancy should be also communicated to women of child-bearing potential as suggested in a recent case report [29].

Some characteristic mutations present in rhodopsin gene may be involved in retinitis pigmentosa, a group of diseases characterized by photoreceptor cell death in the retina and subsequent vision loss. The characterization of mutation may be relevant to evaluating the opportunity for vitamin A treatment in prenatal conditions.

In developed countries, the prevalence rate of vitamin A deficiency (VAD), defined as serum retinol levels lower than 1.05 µmol/L, is estimated to be less than 1% [30], but even in these countries it has been found that there is a substantial proportion of pregnant women of certain ethnic groups who suffer from VAD. Genetic polymorphisms may also contribute to the VAD status differences between ethnic groups.

Three well-identified low-serum retinol single nucleotide polymorphisms (SNPs) have been reported: rs1667255, located between the downstream of the transthyretin (TTR) gene and the downstream of the beta-1,4-galactosyltransferase 6 (B4GALT6) gene, rs10882272, located in the 3′-UTR of the free fatty acid receptor 4 (FFAR4) gene and downstream of the retinol binding protein 4 (RBP4) gene and rs738409 which is located in the coding sequence of the patatin-like phospholipase domain-containing 3 (PNPLA3) gene [31,32]. TTR forms a ternary complex with RBP4 and retinol, transporting vitamin A in the blood to the liver [30].

Suzuki and co-workers evaluated the proportion of lower serum retinol-risk alleles (rs10882272 C and rs738409 G) among different ethnic groups in pregnant women and they found that it is higher in groups with a higher prevalence of VAD (non-Hispanic Blacks and Hispanics/Latinos) and that the risk alleles in homozygosity for both polymorphisms are not frequent in all ethnic groups, the highest rates are present in Mexicans (1.53%) followed by Afro-Caribbeans (0.96%), and the rs738409 G variant in Asians also had a high proportion (0.39). They also found that Asians and non-Hispanic White populations showed high no risk homozygous genotypes (rs738409 C/C and rs10882272 T/T). These results suggest that genetic variants can increase the risk of low-serum vitamin A in non-Hispanic Blacks and Latin Americans with Afro-Caribbean ancestry [30].

### 2.2. Vitamin E

Vitamin E is a lipid-soluble vitamin comprising of four tocopherols and four tocotrienols designated as α-, β-, γ-, and δ-. A recent review reported that vitamin E acts as a chain-breaking antioxidant, preventing the lipid peroxidation spreading [33]. The crucial phase of lipid metabolism and lipoproteins refers to the action of the liver which operates within the mechanisms of oxidation aimed at detoxification. These two main regulatory systems control the state of vitamin E, as well as the preferential hepatic metabolism of non-α-tocopherols.

The RDA of vitamin E can be met by the consumption of plant-based oils (wheat germ, sunflower, safflower), almonds, nuts, sunflower seeds, fruits (mango, avocado), and vegetables (beet greens, collard greens, spinach, pumpkin, pepper), among others (see www.nap.edu accessed on 3 March 2022).

Vitamin E requirements increase by about 25% during late pregnancy (Table 1). Vitamin E serum levels have been found to be lower (9.10 ± 2.47 mg/L) in the first trimester than in the third one (15.80 ± 5.01 mg/L), and excess vitamin E was observed in 5.37% of pregnant women [21]. However, a clinical trial and a review reported that high doses of vitamin E during the first trimester of pregnancy were not associated with a risk of alterations in the newborn, but caused a decrease in birth weight [34,35].

Several pathological conditions occurring during pregnancy, such as hypertensive disorders, placental abortion, miscarriage, and premature birth, as well as aging of the placenta, are associated with vitamin E deficiency [26]. Pregnant women have a fast metabolism leading to increases in free radical production and lipid peroxidation. As a consequence, vitamin E deficiency can be associated with increased free radical production, resulting in placenta aging and vascular endothelial injuries. These effects can be linked to a high incidence of hypertensive disorders in pregnancy, as reported in a recent cross-sectional study and two reviews [17,36,37].

To our knowledge, no data are available regarding the influence of genetics on vitamin E status.

### 2.3. Vitamin D

Vitamin D is involved in bone metabolism by stimulating calcium and phosphate intestinal absorption, and also controls parathyroid hormone (PTH) secretion by negative feedback. Moreover, it also displays other fundamental functions, such as the modulation of immune response, anti-proliferative action, as well as the regulation of insulin production and blood pressure, so that vitamin D deficiency has been independently associated with various pathological states, such as chronic inflammatory diseases, vascular disorders, infective diseases, and neurodegenerative disorders [38,39].

The RDA of vitamin D can be met by the consumption of oily fish (salmon, sardines, herring, mackerel), egg yolk, liver, and red meat (see www.nap.edu accessed on 3 March 2022).

Two recent original articles showed that reference values for calciferol can be influenced by ethnic differences. For example, higher values have been reported in different cohorts of White people rather than in other populations, such as Black pregnant women showing levels <50 nmol/L [40,41].

In a review including thirteen studies from seven countries, the prevalence of vitamin D deficiency ranged from 51.3% to 100% [42]. Poor vitamin D status represents an issue with documented insufficiency in pregnancy in the USA (28%) and in the Mediterranean countries (50–65%) [43].

As previously reported in a notable review, relevant differences in vitamin D and calcium occur depending on vitamin D requirements by mothers to ensure adequate levels of calcium for bone formation [44].

Neonatal hypocalcemia associated with maternal low vitamin D level may be responsible for craniotabes and infant rickets [45]. Children of mothers with low levels of circulating 25-hydroxyvitamin D3 in pregnancy have reduced bone size and density, even in the absence of rachitic skeletal changes [40,41].

Supplementation with a daily dose ranging from 400 to 800 IU of vitamin D is recommended during pregnancy [20] (Table 1). In presence of a higher dose of vitamin D during pregnancy without medical supervision, extra monitoring or treatment is required.

It has also been hypothesized that catabolism of 25(OH)D_3_ to 24,25(OH)_2_D_3_ would be greater in the bolus than in the daily dose group [46].

In order to avoid bone demineralization during lactation, a priority role may be ascribed to both calcitonin and PTH. In particular, bone resorption during lactation appears as an osteoclast-mediated process. The pathway is characterized by low circulating PTH and estradiol, but calcitonin is elevated [47].

Low PTH levels reduce calcium tubular reabsorption, and during pregnancy are associated with urinary stasis secondary to hydronephrosis. These changes promote kidney stone formation in pregnancy [48].

Although a systematic search to demonstrate possible adverse effects associated with vitamin D supplementation in pregnancy was not attempted, some findings documenting toxic events correlated to vitamin D treatment have been reported by Beer and colleagues and in a clinical trial [49,50]. In current medical care, unnecessary vitamin D administration is considered bad practice. Unfortunately, current evidence on adverse effects only derives from short-term follow-up studies. Generally, toxic effects are associated with vitamin supplements at high doses (greater than 10,000 IU of vitamin D) [49,50]. In this regard, to reduce side effects several attempts have been carried out by synthesizing vitamin D analogues [51,52]. Furthermore, a review summarizing experimental data on tumor-suppressive effects of natural agents including vitamin D, reported that using high doses of calcitriol can stimulate its antiproliferative effects leading to only transient hypercalcemia [53].

Paradoxically, a greater loss of bone mineral density has been ascribed to excess vitamin D supplementation with 4000 IU or 10,000 IU, in comparison with 400 IU daily, in healthy vitamin D-sufficient females, but not males [54].

An increased risk of poor pregnancy outcomes has been observed in subjects with low 25(OH)D3 levels, and in the preconception period, a 25(OH)D3 cut-off level higher than 37.5 nmol/L has been suggested to prevent adverse pregnancy outcomes [55].

It is interesting to note that another review reported that during pregnancy, gene transcription regulation as well as other physiological processes can be influenced by vitamin D supplementation [56]. More recently, it has been demonstrated that offspring health may be correlated with vitamin intake during pregnancy. Evidence was given for association between inadequate or high intakes of folic acid and vitamin D and good or poor neurocognitive development of the offspring [57].

Recent results from a genome-wide association study suggested that the impact of pregnancy- and birth-related physiological modifications on vitamin D concentrations can be influenced both by genetic alterations in vitamin D metabolic pathways and by the activity dependent on specific loci involved in the immune function [58].

The nuclear vitamin D receptor (VDR) is involved in numerous functions in physiologic pregnancy, such as implantation, placental immune modulations, and hormone secretion.

In another genome-wide association study, changes in vitamin D levels have been associated with multiple loci in VDR binding proteins and the modulating enzymes CYP2R1, CYP24A1, CYP27B1, and CYP27A1 genes. The authors stated that these genetic variants identify individuals at elevated risk for vitamin D deficiency [59].

Four di-allelic VDR gene polymorphisms have been described: BsmI (A > G, rs1544410), ApaI (A > C, rs7975232), FokI (C > T, rs10735810), and TaqI (T > C, rs731236) [60,61].

The FokI genotype has been associated with the modification in the offspring size whereas the ApaI noncoding polymorphism (rs7975232) and TaqI polymorphism (rs731236) are associated with variation in mRNA stability [60].

It must be considered that the correlation between fetal growth restriction and a maternal polymorphism may vary depending on ethnic group. In fact, in a cohort study conducted in North Carolina, ApaI (rs7975232) was correlated with offspring size in Black mothers but not in White ones [62].

With regard to the possible correlation between VDR polymorphisms and the risk of spontaneous preterm birth, Rosenfeld et al. investigated FokI, ApaI, TaqI, and BsmI SNPs in an Israeli population. The authors found that women carrying the ApaI homozygote genotype had a higher risk for preterm births compared to the heterozygote group but no correlation has been found between VDR BsmI, TaqI, or FokI variation frequencies and preterm and term births [63]. On the contrary, Manzon et al. found that an increased risk of preterm birth could be related to maternal FokI variant (odds ratio OR = 3.317) [62]. A study conducted on Polish population showed that TaqI, BsmI, and ApaI polymorphisms, had no effect on preterm birth [64].

Several studies found a correlation between VDR polymorphisms and a higher risk for type 1 and type 2 diabetes mellitus (T1DM and T2DM) [65,66,67].

A recent meta-analysis showed an increased susceptibility to gestational diabetes mellitus (GDM) associated with VDR ApaI (rs7975232) and FokI (rs2228570) polymorphisms. The VDR BsmI (rs1544410) polymorphism was linked with GDM in Asian and African population whereas the VDR TaqI (rs731236) polymorphism was not associated with GDM [68].

A correlation between VDR ApaI, TaqI, and FokI SNPs and the risk for GDM was indicated in Iranian, Saudi Arabian, and Chinese populations [69]. In the latter cohort, the FokI polymorphism was significantly related to T2DM risk, unlike in White people [70]. Moreover, the genetic variants of the group-specific component protein gene (GC) rs16847024 C > T, and retinoic X receptor gene (RXR), RXRG rs17429130 G > C and RXRA rs4917356 T > C were found to be significantly involved in the enhanced risk of GDM [71].

No association has been found between FokI, ApaI, and BsmI SNPs and preeclampsia or gestational hypertension [72], despite the strong correlation between these VDR polymorphisms and hypertension risk not in pregnancy [73,74,75]. However, a case-control study showed that the VDR FF/bB haplotype resulted in a double increase to the risk for gestational hypertension in vitamin D-deficient pregnant women [76].

In light of these observations, a genotype analysis for VDR polymorphisms in pregnant women may be useful for an ad hoc vitamin D supplementation strategy.

A study from Southern Europe showed that maternal and neonatal vitamin D-binding protein (VDBP) polymorphisms do not influence neonatal vitamin D status at birth, whereas, in a maternal cohort with no vitamin D supplementation during pregnancy, mothers with CC genotype for rs2298850 and CC genotype for rs4588 demonstrate higher 25-hydroxyvitamin D concentrations (≥75 nmol/L) during delivery [77].

### 2.4. Vitamin K

Vitamin K (VK) is involved in blood coagulation and its deficiency can result in haemorrhage. VK is considered critical for pregnant women and newborns because of poor placental transport and low concentrations of VK in breast milk [22].

The RDA of vitamin K can be met by the consumption of green leafy vegetables (spinach, cabbage, kale, Brussels sprouts, collard and turnip greens, lettuces, broccoli), soybean oil, and, to a lesser extent, eggs, cheese, and meat, among foods (see www.nap.edu accessed on 3 March 2022).

VK intake is equal to 90 µg/day under physiological conditions and during pregnancy (Table 1).

Shahrook S and colleagues in their systematic review and metanalysis found that the effect of prenatal VK was not statistically significant for neonatal bleeding reduction with an exception for maternal plasma VK1, but further investigations are needed [78].

There is no proven toxicity regarding the vitamin K natural form. In fact, the review by Shearer claims that the rare reports of toxicity concern components for injectable and parenteral formulations other than vitamin K such as the solubilization vehicle [22].

Toxic effects, i.e., allergic reactions, hemolitic crises, and hepatotoxicity, were reported for vitamin K3 synthetic that was banned from over-the-counter sales in the US [79]. For more details, see the very recent publication by Imbrescia and Moszczynski [80].

Vitamin K–dependent clotting factor deficiency (VKCFD) is a rare inherited form of defective γ-carboxylation resulting in the early onset of bleeding. Two variants of this autosomal recessive disorder have been identified: VKCFD1 and VKCFD2. The first one is associated with point mutations in the γ-glutamyl-carboxylase gene (GGCX), and the second one derives from point mutations in the vitamin K epoxide reductase gene (VKOR) [81].

VKCFD occurs rarely and only missense mutations have been identified suggesting embryonic lethality with complete deficiency of either enzyme in humans [82].

Vitamin K treatment can improve partially Factors II, VII, IX, and X activity levels in VKCFD patients so genotyping for VKORC and GGCX is strongly recommended to avoid multiple and/or severe bleeding episodes [81].

Growth arrest-specific 6 (Gas6), a member of the vitamin K-dependent protein family, binds all three receptor tyrosine kinases of the TAM (Tyro-3/Axl/Mer) to perform several biological functions. In a recent review, Gas6 has been suggested as a novel atherothrombotic risk factor with anti-angiogenic and pro-atherogenic properties [83]. It has been shown that plasma Gas6 concentrations were significantly lower in patients with preeclampsia (PE) as compared to normotensive pregnant (NP) women. The authors found that polymorphism of GAS6 c.834 + 7G4A in intron 8 was significantly associated with PE, and allele A of the GAS6 c.834 + 7G4A polymorphism seems to be associated with a reduced risk for PE [84].

## 3. Relevant Findings on Water-Soluble Vitamins

The most relevant literature data on critical issues related to the supplementation of water-soluble vitamins in pregnant women are discussed in the following paragraphs.

Normal reference levels and recommended intake, when available, of each vitamin in different physiological conditions, have been derived from literature data, DRI reports, and Institutes of Medicine National Academy (see www.nap.edu accessed on 3 March 2022), and are shown in Table 2.

### 3.1. Folic Acid

Folic acid (vitamin B9) is an essential nutrient required for the synthesis of nucleic acids and heme group, and for protein metabolism. Thus, it is critically needed during periods of rapid growth, such as during pregnancy and fetal development.

Foods that are good sources of folate are dark green leafy vegetables (spinach, broccoli, romaine lettuce, turnip greens, asparagus, Brussels sprouts), fresh fruits, beans, peanuts, sunflower seeds, eggs, liver, whole grains, and seafood, among foods (see www.nap.edu accessed on 3 March 2022).

Vitamin B9, together with a multivitamin supplement, during pregnancy or in women of reproductive age, may be useful to reduce occurrence of birth defects. In Western society, vitamin B9 intake may be sufficient. Instead, in some population folate-multivitamin supplementation can be necessary and should be assumed once daily. The recommended intake in early and late pregnancy is increased by 50% and 25%, respectively, compared with normal requirement (Table 2).

Vitamin B9 supplementation during pregnancy is known to protect the fetus from neural tube defects. Furthermore, tetra-hydrofolate plays an important role in the metabolism of the one-carbon units involved in the epigenetic regulation of transcription which underlies development. Therefore, unexpected persistent effects in the offspring may be caused by maternal folic acid supplementation, as suggested in a review [89]. A more recent review claims that, although fortification of grains with vitamin B9 may be useful for public-health outcome, further investigations are needed to ascertain if excessive folic acid intakes increasing methyl group availability may have potential hazardous effects on newborns [90]. In addition, a case report described small intestine perforation after ingestion of a high amount of vitamin B9 supplements [91].

Vitamin B9 works closely with vitamin B12 in the re-methylation cycle that produces methionine from homocysteine (Hcy) [90]. Vitamin deficiency leads to elevated homocysteine levels, namely hyperhomocysteinemia, that has been identified as an independent risk factor for the development of atherosclerosis and other inflammation- and oxidative stress-related conditions.

Recent data suggested that suitable approaches for the prevention of exceeding folate intake may critically depend on the underlying mechanisms of biochemical defects. Folate status and hyperhomocysteinemia usually are determined by poor dietary intake, unhealthy lifestyle (i.e., alcohol excess), and the presence of common genetic variants, such as methylenetetrahydrofolate reductase (MTHFR) C677T and A1298C polymorphisms [92].

Several studies focused on the association between SNPs of MTHFR gene and increased risk of adverse birth outcomes [93,94,95,96,97].

However, intervention with folic acid alone may not only be inefficient, but even cause harm to women living in regions where vitamin B12 deficiency is endemic [98].

A common polymorphism in the MTHFR gene is represented by cytosine to thymine substitution at position 677 in exon 4 (MTHFR C677T). This substitution leads to the insertion of valine for alanine in the corresponding protein resulting in a reduction in enzyme activity [99] and accumulation of plasma Hcy [93].

MTHFR polymorphisms are known to reduce the bioavailability of folate and have been linked to pregnancy complications such as preeclampsia (PE) and intrauterine growth restriction (IUGR) [100,101,102].

A meta-analysis performed by Chen et al. in 2016, by considering studies published before August 2014, found that there was no association of the MTHFR C677T polymorphism with pre-term birth (PTB) or placental abruption [93].

A recent meta-analysis evaluated both the maternal and neonatal MTHFR C677T polymorphism confirming that there is a conclusive association between maternal MTHFR C677T polymorphism and preterm birth (PTB) as well as low birth weight (LBW) risk and no significant association between neonatal MTHFR C677T polymorphism with PTB or LBW [103].

Based on development status, this meta-analysis showed that there is a statistically significant association between the maternal MTHFR C677T polymorphism and PTB as well as LBW risk in developing countries in comparison with results obtained in studies from developed countries. These findings are not surprising as pregnant women in developing countries might not intake folate adequately as opposed to their counterparts in developed countries [104].

Moreover, it is well-known that women with the MTHFR 677TT genotype are predisposed to increased plasma homocysteine levels when folate intake is insufficient; thus pregnant woman with TT genotype in developing countries are more likely to give birth to babies with PTB or LBW [105].

It has been found that MTHFR 1298CC was associated with increased risk for PE, whereas there was an association between the maternal transcobalamin 2 (TCN2) 776GG genotype and a decreased risk for spontaneous preterm births [106].

TCN2 polymorphism (C > G substitution) alters the structure of the TCN2 protein modifying its affinity for vitamin B12, resulting in an easier release from the bound TCN2 transporter with a consequent reduced vitamin B12 transport into cells and thus higher Hcy levels compared with the CC genotype. The authors showed that folic acid supplementation leads to higher serum folate and vitamin B12 concentrations, a reduced uterine artery resistance index, and increased birth weight [107].

Higher circulating Hcy levels were also found in pregnant woman with the MTHFD1 G1958A mutation, which can result in a reduction in the stability of its synthetase domain, modifying its metabolic activity to limit the availability of methyl THF [108]. The AA genotype is also associated with neural tube defects and cardiac malformations in humans as MTHFD1 enzyme function is involved in purine synthesis and pregnancy outcome [109].

Different approaches for the prevention or reduction of congenital anomalies should take into account the age of subjects, ethnicity, compliance, and genetic risk conditions [110,111].

### 3.2. Vitamin B12 and Vitamin B1

Vitamin B12 is an essential micronutrient, naturally present in eggs, fish, meat, poultry, and dairy products, and only in small amounts in vegetables. Other than in Hcy metabolism, vitamin B12 is involved in the one-carbon units metabolism related to synthesis and stabilization of nucleic acids as well as in DNA methylation, required for the epigenetic regulation of gene expression. Moreover, vitamin B12 is needed for erythropoiesis as well as for development and myelination of nerve cells (see www.nap.edu accessed on 3 March 2022).

The recommended intake of vitamin B12 during pregnancy ranges from 2.6 to 2.8 µg/day (Table 2). Vitamin B12 deficiency may be a cause of infertility or recurrent spontaneous abortion and an inadequate vitamin B12 status at the beginning of a pregnancy may increase the risk of birth defects, such as neural tube defects [112].

To our knowledge, no literature data are available regarding the influence of genetics on vitamin B12 status.

Vitamin B1, also known as thiamine, is a micronutrient with multiple functions in carbohydrate metabolism. Its deficiency leads to an impaired oxidative and energy metabolism, causing serious and potentially irreversible neurological damage or death [87].

The RDA of vitamin B1 can be met by the consumption of yeast, meat (beef, pork), liver, whole grains, germ of cereals, eggs, as well as fruits (oranges) and vegetables (pulses, kale, asparagus, cauliflower, potatoes).

It is well known that pregnancy and lactation require an increased thiamine need. The analysis of vitamin B1 concentrations showed that approximately 50% of women develop a biochemical thiamine deficiency during the trimesters of pregnancy [113]. The deficiency occurs particularly when dietary intake is inadequate or excessive alcohol is consumed, as reported in a recent review [114].

During pregnancy, thiamine requirements increase, probably as result of the vitamin sequestration by the fetus and placenta (Table 2).

In fact, thiamine and other water-soluble vitamins (B9, B12, and C) are 2–5 times more concentrated in the umbilical cord blood than in maternal blood [115]. Moreover, Ortega and colleagues confirmed that maternal thiamine intake influences mature breast milk thiamine concentration and the activation coefficient of erythrocyte transketolase (a-ETK) [116].

Regarding vitamin B1 toxicity, there is no determined tolerable upper intake level for thiamine. Thiamine transporters 1 (THTR1) encoded by the gene solute carrier family 19 member 2 (SLC19A2) and thiamine transporters 2 (THTR2) encoded by solute carrier family 19 member 3 (SLC19A3), perform the function of carrying thiamine to the cells. The enzyme thiamine pyrophosphokinase (TPK1) activates thiamine within the cell, forming thiamine diphosphate (TDP), a cofactor for several enzymes involved in the regulation of glucose metabolism. It is known that mutations in SLC19A2 and SLC19A3 are rare and they cause monogenic diseases, whereas variants in TPK1 are common and are associated with birth weight [117,118,119]. Bartáková et al. found that transketolase (TKT) activity, which requires vitamin B1 as a cofactor, in the postpartum depend on the genotypes of SLC19A2 SNP rs6656822 and SLC19A3 SNP rs7567984; the enzymatic activity is higher in homozygotes TT for the first one and in heterozygotes CT for the second one. The authors suggested that inter-individual variability in thiamine metabolism may be involved in the postpartum persistence of glucose and in the susceptibility to GDM [117].

### 3.3. Vitamin C

Vitamin C is a powerful antioxidant needed for regeneration of other antioxidants, such as vitamin E. Its antioxidant action has been reported to be protective against the development of oxidative stress-related disorders, such as cancer and cardiovascular diseases. Vitamin C also plays a key role in many biosynthetic pathways, i.e., synthesis of collagen and neurotransmitters, in immune function and in iron absorption by intestine. Vitamin C deficiency is associated with the development of scurvy, capillary fragility, and abnormal wound healing [119].

The RDA of vitamin C can be met by the consumption of fresh fruits (oranges, kiwi, lemon, strawberries, grapefruit) and vegetables (tomatoes, bell peppers, cabbage, broccoli, cauliflower, white potatoes), among foods (see www.nap.edu accessed on 3 March 2022).

On the basis of recent investigation, a role for vitamin C supplement in order to avoid preeclampsia, intrauterine growth restriction and maternal anemia has been proposed. Data analysis in a prospective cohort study involving 24,300 women clearly demonstrated that a vitamin C supplement is effective for the prevention of fetal or neonatal death, poor fetal growth, and preterm birth or pre-eclampsia [120].

For most people, the recommended doses of vitamin C are likely safe (Table 2); however, vitamin C at daily doses higher than 2000 mg might cause some adverse effects, such as vomiting, heartburn, and stomach cramps [121].

Taking into account the widespread use of vitamin supplements during pregnancy, the values of recommended intake should be indicated in a range of tolerable doses.

In an observational study conducted in North Carolina in 2064 women, low vitamin C intake, either before conception or during the second trimester, was associated with an elevated risk of preterm, premature rupture of the membranes. Women with low vitamin C intake during both time periods showed the greatest risk [122].

These observations suggest that genetic variants in vitamin C transport could contribute to the risk for preterm birth-related outcomes.

An electronic letter reported that vitamin C is transported by one of two sodium-dependent vitamin C transporters (SVCT1, encoded by SLC23A1 and mapped to 5q31.2; and SVCT2, encoded by SLC23A2 and mapped to 20p13) [123].

Erichsen and colleagues showed that three SLC23A2 individual SNPs were associated with preterm birth in White individuals, but after hierarchical regression analysis, only SLC23A2-08 (an intron 2 variant, rs6139591), was strongly associated. The authors found that individuals heterozygous for the T allele of SLC23A2-08 showed some elevation in risk, whereas individuals homozygous for the T allele had a nearly threefold elevated risk of preterm delivery [124].

Duarte-Salles et al. found a correlation between genotoxic airborne polycyclic aromatic hydrocarbons (PAH) benzo(a)pyrene [B(a)P] intakes and significant reduction both in birth weight and length, and an increase in risk of size for gestational age (SGA) among women with low dietary vitamin C intakes during the first trimester of pregnancy.

Among these women, associations were strongest in those carrying the glutathione S-transferase P1 (GSTP1) Ile105Val polymorphism, that is associated with lower contaminant detoxification activity. On the contrary, no association was found between dietary B(a)P and SGA among women with high vitamin C intake (OR: 0.81; 95% CI: 0.23–2.75). The authors did not observe significant interactions with vitamin E, alpha-carotene or beta-carotene intakes in associations between dietary B(a)P and fetal growth indicators [125].

### 3.4. Other Water-Soluble Vitamins

Few data are available regarding the role of other water-soluble vitamins during pregnancy. The requirement for some vitamins, such as nicotinamide (B3, niacin), riboflavin (B2), and biotin (vitamin B7) is, in most cases, ensured by the diet in developed countries. Adequate amounts of riboflavin and niacin are provided by enriched cereals and grain-containing products, whereas biotin can be mainly found in foods like eggs, milk, and bananas [126]. These vitamins act as co-factors of enzymes involved in the metabolism of lipids, carbohydrates, and other substrates, in energy production, and DNA repair, and can be also synthesized by intestinal bacteria [127].

Vitamin B6, in its main form pyridoxal phosphate, acts as a co-factor for numerous enzymes, involved in the catabolism of proteins, carbohydrates, and lipids, in homocysteine trans-sulfuration to cysteine, and in immune function and brain health. Six pyridine derivatives related to phosphorylation are indicated as vitamin B6.

The RDA of vitamin B6 can be met by the consumption of fish (salmon, tuna), meat (beef liver, poultry), legumes (chickpeas), some vegetables (dark leafy greens), and fruits (bananas, oranges, cantaloupe, papayas), among others (see www.nap.edu accessed on 3 March 2022).

Vitamin B6 supplementation influenced nausea and vomiting, and produced beneficial effects in dental health. In a meta-analysis based on three small studies, it has been reported that vitamin B6 supplementation had a significant positive effect on birth weight [128].

Vitamin B6 decreases physiologically during pregnancy probably due to the increase in blood volume. Perhaps this is why its deficient status has not been indicated in pregnant women with reduced parameter conditions [129].

## 4. Conclusions

To the best of our knowledge, available research findings about the general requirement of different vitamins during pregnancy had not yet been summarized so far. This is the first narrative review providing an overview of the issues related to the inappropriate use of vitamin supplements during pregnancy, and also discussing the influence of genetic background on the individual susceptibility to adverse events consequent to vitamin supplementation.

The physiological need for some nutrients and food groups is regulated both by nutritional deficiencies and genetic predisposition to various dysfunctions. The relationship between nutrition and health is somewhat stronger for dietary models, such as the Mediterranean-type diet, than for individual nutrients. This emphasizes the beneficial effects of the overall presence of various compounds in these diets. Several studies so far have explored the role of maternal vitamins in fetal growth, including brain development. However, further investigation is needed to define vitamin impact when the intake during pregnancy is not only inadequate, but may even exceed the requirements. In fact, given the known beneficial effects of vitamins, the administration of high vitamin doses has become a widespread trend, increasing the risk of toxic effects. Moreover, it should be emphasized that the presence of maternal genetic variants affecting vitamin signaling pathways can increase the risk for the development of unfavorable pregnancy outcomes.

The use of advanced technologies for the overall assessment of nutritional status and the availability of new nutritional biomarkers will be helpful to maintain optimal physiological conditions. In particular, the evaluation of genetic variants may be an essential tool with which to establish the association between vitamin status and risk factors of some pathological conditions during pregnancy. The study of genetic and epigenetic characteristics should represent the topic of future research.

## Figures and Tables

**Table 1:** Normal reference levels and recommended intake of fat-soluble vitamins A, E, D, and K in different physiological conditions.

Fat-Soluble Vitamins	Reference Levels	Recommended Intake	Recommended Intake in Early Pregnancy	Recommended Intake in Late Pregnancy	References
**Vitamin A**	0.2–0.6 mg/L	700 µg/day	770 µg/day	1300 µg/day	[17,18]
**Vitamin E**	7.4–23.0 mg/L	15 mg/day	15 mg/day	19 mg/day	[17]
**Vitamin D**	75–200 nmol/L	15 µg/day	25 µg/day	35 µg/day	[19,20]
**Vitamin K**	0.09–1.99 nmol/l	90 µg/day	90 µg/day	-	[21,22]

**Table 2:** Plasma levels and reference values for the intake of water-soluble vitamins B9, B12, B1, and C.

Water-Soluble Vitamins	Plasma Levels	Nutrient Intake under Physiological Conditions	Recommended Intake in Early Pregnancy	Recommended Intake in Late Pregnancy	References
**Vitamin B9**	2.2–17 ng/ml	400 µg/day	600 µg/day	500 µg/day	[85]
**Vitamin B12**	2–9 µg/ml	2.4 µg/day	2.6 µg/day	2.8 µg/day	[86]
**Vitamin B1**	5–12 μg/dL	1.2 mg/day	1.4 mg/day	-	[87]
**Vitamin C**	6–14 mg/L	75 mg/day	85 mg/day	120 mg/day	[88]

## Data Availability

Not applicable.
